# Comprehensive analysis of cucumber *RAV* family genes and functional characterization of *CsRAV1* in salt and ABA tolerance in cucumber

**DOI:** 10.3389/fpls.2023.1115874

**Published:** 2023-02-02

**Authors:** Jialin Li, Chunying Song, Hongmei Li, Siqi Wang, Linyue Hu, Yanlei Yin, Zenghui Wang, Wenxing He

**Affiliations:** ^1^School of Biological Science and Technology, University of Jinan, Jinan, China; ^2^Xilin Gol League Agricultural and Animal Product Quality and Safety Monitoring Center, Xilinhot, China; ^3^Shandong Institute of Pomology, Tai’an, Shandong, China

**Keywords:** abiotic stresses, cucumber, expression patterns, RAV family, salt tolerance, transcription factor

## Abstract

The RAV (related to ABI3 and VP1) transcription factors are specific and exist in plants, which contain a B3 DNA binding domain and/or an APETALA2 (AP2) DNA binding domain. *RAVs* have been extensively studied in plants, and more and more evidences show that *RAVs* are involved in various aspects of plant growth and development, stress resistance and hormone signal transduction. However, the systematic analysis of *RAV* family in cucumber is rarely reported. In this study, eight *CsRAV* genes were identified in cucumber genome and we further comprehensively analyzed their protein physicochemical properties, conserved domains, gene structure and phylogenetic relationships. The synteny analysis and gene duplications of *CsRAV* genes were also analysed. *Cis*-element analysis revealed that the *CsRAVs* promoter contained several elements related to plant hormones and abiotic stress. Expression analysis showed that NaCl and ABA could significantly induce *CsRAV* genes expression. Subcellular localization revealed that all CsRAVs were localized in the nucleus. In addition, *35S*:*CsRAV1* transgenic *Arabidopsis* and cucumber seedlings enhanced NaCl and ABA tolerance, revealing *CsRAV1* may be an important regulator of abiotic stress response. In conclusion, comprehensive analysis of *CsRAVs* would provide certain reference for understanding the evolution and function of the *CsRAV* genes.

## Introduction

The *RELATED TO ABI3/VP1* (*RAV*) family belongs to one of the plant-specific *B3* superfamily, which also contains three other families encompassing the auxin response factor (*ARF*) family, leafy cotyledon2 (*lec2*)-abscisic acid insensitive3 (*abi3*)-val (*LAV*) family, and reproductive meristem arf (*REM*) family (Swaminathan et al., 2008; Wang et al., 2012a). All members of the *B3* superfamily contain a region of about 110 amino acids called the B3 domain, which is a DNA binding domain named because it is the third basic domain in the maize gene *VIVIPAROUS1* (*VP1*) (Kagaya et al., 1999). Most *B3* genes in the *ARF* and *LAV* families have been extensively studied, but *B3* genes in the *RAV* family are rarely unknown. The RAV proteins contain B3 domain and/or AP2 (APETALA2) domain (Lu et al., 2014). Therefore, *RAV* family members can reasonably be classified as *B3* superfamily members or *AP2/EREBP* family members (Matías-Hernández et al., 2014). In *Arabidopsis*, there are 13 *RAV* genes, of which seven contain only the B3 domain and the other six contain both B3 and AP2 domains (Magnani et al., 2004; Romanel et al., 2009; Fu et al., 2014). Of these, *AtRAV1* and *AtRAV2*, containing both B3 domain and AP2 domain, were the earliest discovered members of the *RAV* family (Kagaya et al., 1999).

Increasing evidence has shown that RAV transcription factors play key roles in a few aspects of plant growth and development. *Arabidopsis RAV1* negatively regulates flowering time, hypocotyl elongation, and seed development (Hu et al., 2004; Woo et al., 2010; Shin and Nam, 2018). Similarly, ectopic expression of soybean *RAV1* in tobacco results in slow development and delayed flowering time (Zhao et al., 2008; Lu et al., 2014). It was reported that members of *RAV* family *TEMPRANILLO 1* and *2* (*TEM1* and *TEM2*) negatively regulate flowering time by inhibiting *FLOWERINGLOCUS T* (*FT*) expression and the production of gibberellins in *Arabidopsis* (Castillejo and Pelaz, 2008; Marín-González et al., 2015; Kabir et al., 2021). The *NGATHA* genes (*AtNGA1*-*AtNGA4*), members of the *RAV* family in *Arabidopsis thaliana*, have been shown to play key roles in lateral organs development. Over-expression of *AtNGA1* to *AtNGA4* present small and narrow leaf and flower, whereas the *nga1/nga2/nga3/nga4* quadruple mutant reveals large and wide lateral organs (Alvarez et al., 2009; Lee et al., 2015). Besides, overexpressing of *Brassica rapa NGA1* (*BrNGA1*) in *Arabidopsis thaliana* gives rise to significantly smaller and narrower lateral organs such as roots, flowers, leaves and cotyledons due to a reduction in cell numbers compared to the wild type (Kwon et al., 2009). In rice, *OsRAV9*/*OsTEM1* was identified as a negative regulator of floral transition. Moreover, the down-regulation of *OsRAV11* gene was associated with ovary enlargement and seed weight increase (Osnato et al., 2020). The results of functional characterization of RAVs support the notion that RAVs are negative growth regulators in plant (Shin and Nam, 2018).

It is also reported that RAV transcription factors are involved in biotic and abiotic stress responses, such as salt, drought, plant pathogens and plant hormones. The ectopic expression of soybean *GmRAV3* in *Arabidopsis thaliana* can improve the resistance of transgenic lines to high salt and drought, and lead to the insensitivity of transgenic plants to exogenous ABA (Lu et al., 2014; Zhao et al., 2017). The *OsRAV2* expression can be significantly induced by salt in rice, indicating its important roles in salt response (Duan et al., 2016). Melon *RAV1* (*CmRAV1*) can be induced by NaCl treatment and ectopic overexpression of *CmRAV1* in *Arabidopsis* enhances salt tolerance at the seed germination and seedling stages (Zhao et al., 2019a). It has been reported that MeRAV1 and MeRAV2 play important roles in resistance to cassava bacterial blight through activation of melatonin biosynthesis genes in cassava (Wei et al., 2018). In addition, over-expression of tomato *RAV2* (*SlRAV2*) gives rise to increased bacterial wilt (BW) tolerance, whereas knockdown *SlRAV2* significantly decreases tomato resistance to BW (Li et al., 2011). Meanwhile, members of the *RAV* family can be induced by some plant hormones and have been shown to be involved in brassinosteroid and ethylene responses (Alonso et al., 2003; Hu et al., 2004). The expression of all soybean *RAV* genes increases dramatically under ABA treatment (Zhao et al., 2017). In cotton, *GhRAV4*, *GhRAV9* and *GhRAV20* genes are significantly induced by BL, JA and IAA hormones (Kabir et al., 2021). Therefore, it clearly shows that members of the *RAV* gene family play important roles in the development and stress response of different plant species.

Cucumber (*Cucumis sativus* L.) is one of the most important vegetable crops in the world (Huang et al., 2009). Despite the important roles of *RAV* genes in plant growth, development and stress tolerance, the functions of *CsRAVs* in cucumber are largely unknown. In this study, we identified eight *CsRAV* genes and classified them into four clades. The gene structures, conserved motifs, phylogenetic analysis, synteny analysis and gene duplications were further performed. In addition, the expression patterns of *CsRAV* genes under different abiotic stresses were also measured. Furthermore, overexpression of *CsRAV1* increased salt tolerance and ABA resistance. These results lay a foundation for the evolutionary and functional characterization of *RAV*s in cucumber.

## Materials and methods

### Genome-wide identification of *CsRAVs* in cucumber

To identify the *CsRAV* genes from cucumber (Chinese Long) v3 genome database (http://cucurbitgenomics.org/organism/20), 13 AtRAV proteins were used as query sequences and Blastp was used to search for the predicted cucumber proteins. All candidate genes were further confirmed by the existence of B3 (PF02362.21) and/or AP2 (PF00847.20) domains using the Pfam and Simple Modular Architecture Research Tool (SMART) datebase (http://smart.embl-heidelberg.de). The RAV protein sequences in *Arabidopsis* and tomato were downloaded from the *Arabidopsis* Information Resource database (https://www.Arabidopsis.org) and the Solanaceae Genomics Network (https://solgenomics.net), respectively.

### Phylogenetic analysis, conserved motif and gene structure analysis

A phylogenetic tree containing 115 RAV protein sequences from ten species and a phylogenetic tree with the full-length amino acid sequences of nine SlRAVs, 13 AtRAVs and eight CsRAVs were constructed using MEGA 7.0 respectively and the neighbour-joining (NJ) method was used with the following parameters: Poisson correction, pairwise deletion, and bootstrap (1000 replicates; random seed) (Li et al., 2022). The corresponding cDNA and DNA sequences of *RAV* genes were downloaded from corresponding genomes, and the gene structures were analyzed as described by Li et al. (2022). The conserved motifs in RAVs were identified using Multiple Expectation Maximization for Motif Elicitation (MEME) online program (http://meme-suite.org/index.html). *CsRAV* genes were classified according to its phylogenetic relationship with *RAVs* in other species. The visual evolutionary tree, conserved motif and gene structure maps were completed using TBtools (Li et al., 2020).

### Chromosomal distribution and gene duplication

All *CsRAV*s were mapped to cucumber chromosomes based on physical location information from the database of cucumber genome using Circos (Krzywinski et al., 2009). Multiple Collinearity Scan toolkit (MCScanX) was used to analyze the gene duplication events, with the default parameters (Wang et al., 2012b). To show the synteny relationship of the orthologous RAVs in cucumber, *Arabidopsis* and tomato, we used TBtools to constructed the syntenic analysis maps (https://github.com/CJ-Chen/TBtools) (Chen et al., 2020).

### *Cis*-element analysis on *CsRAVs* promoter in cucumber

The relevant data of cucumber genome were downloaded from cucumber genome database (Chinese Long 9930: http://cucurbitgenomics.org/), and we used TBtools to extract the 2 kb sequence of *CsRAVs* gene promoter. The *cis*-elements on the promoter regions of *CsRAV* genes were analysed using PlantCARE website (http://bioinformatics.psb.ugent.be/webtools/plantcare/html/) (Li et al., 2020).

### Vector construction, transient transformation of cucumber cotyledons and *Arabidopsis* transformation

The coding sequence of *CsRAVs* was recombined into pCAMBIA1300 vector with a GFP tag to obtain *35S*:*CsRAVs*-GFP. The *35S*:*CsRAV1*-GFP construct was transformed into *Agrobacterium tumefaciens* LBA4404, and then transferred into *Arabidopsis* (Col-0) or 8-d-old cucumber cotyledons (Li et al., 2020). The homozygous T_3_ transgenic *Arabidopsis* lines were screened and identified for subsequent experiments. The primers used are listed in **Supplementary Table 5**.

### Subcellular localization of CsRAVs

Tobacco leaf epidermal cells were injected with the empty GFP vector and the *35S*: *CsRAVs*-GFP recombinant plasmids, respectively. Then the injected tobaccos were grown under normal conditions for about 48 h, the subcellular localization of CsRAVs were determined by observing the fluorescence signal under a fluorescence microscope.

### Analysis of *CsRAV* gene expression under different abiotic stresses

The cucumber inbred line (Xintaimici) was used as materials to undergo stress treatments and transient transformation. The leaves of vigorous two-week-old cucumber seedlings were sprayed with 100 mM NaCl and 100 μM ABA, respectively. Leaves were taken at 0, 0.5, 1, 3, 6, 9, 12 and 24h for quantitative analysis. The two-week-old cucumber seedlings were cultured in a 4°C incubator with 16 h light/8 h dark for low temperature treatment, and sampled at the same time point for storage. Each sample was taken from six plants and each treatment had three biological replicates.

### Transient transformation of cucumber cotyledons

The coding sequence of *CsRAV1* was recombined into the pCAMBIA1300 vector (universal vector: stored in our laboratory), which was then transformed into *Agrobacterium tumefaciens* LBA4404. The *A. tumefaciens* LBA4404 cells containing the recombinant vector was incubated in liquid medium overnight until the optical density (600 nm) was about 0.6-0.8. Then the *Agrobacterium* solution was centrifuged at 8000 × g for three min, and re-suspended with MES solution (consisting of 10 mM MES, 10 mM MgCl_2_ and 200 μM Acetosyringone) to OD600 value of 0.6-0.8. The suspensions containing the target gene were injected into the cotyledons of 6-7-d-old cucumber seedlings with a 1ml disposable syringe, and cultured in the dark at 20°C for one day, then the follow-up related treatment experiments were conducted.

### Tolerance of transgenic plants to abiotic stress

The seeds of *35S*:*CsRAV1* T_3_-generation homozygous lines and WT were seeded in vermiculite soil and cultured at 22°C for 3 weeks under normal conditions. For salt treatment, the 3-week-old seedlings were irrigated with 200 mM NaCl solution every two days, and the growth of different lines was observed every three days. Under ABA treatment, the transgenic lines and WT were watered 100 μM ABA solution every two days, respectively, and phenotypic evaluation was performed every three days. To investigate the seed germination rate of transgenic and WT seeds under salt stress and ABA treatment, the seeds of transgenic lines and WT were sown on 1/2 MS medium containing 100 mM NaCl or 2 μM ABA, respectively, and cultured under normal conditions. Germination rate was measured after 7 days of culture. The cotyledons of 8-d-old transgenic cucumber seedlings with transient transformation of *35S* and *35S*:*CsRAV1* were subjected to salt and ABA tolerance by hydroponic method. Transgenic cucumber seedlings with equivalent growth were selected and transferred to 2 L Hoagland nutrient solution for hydroponics. After two days of hydroponics, NaCl and ABA were added into the nutrient solution, and the final concentrations of NaCl and ABA in nutrient solution were 100 mM and 100 μM, respectively. To obtain accurate experimental results, the cucumber seedlings transfected with *35S* and *35S*:*CsRAV1* were cultured in the same tank. The phenotypes of transgenic seedlings and control seedlings were observed at different periods (Li et al., 2020).

## Results

### Identification and analysis of *RAV* Genes in cucumber

To identify the putative *CsRAV* family genes in cucumber genome, BlastP was used to search against cucumber genome database based on 13 *Arabidopsis* RAV proteins and consensus protein sequences of B3 (PF02362) and AP2 (PF00847). A total of eight *CsRAVs* were identified in the cucumber genome and the presence of the B3 and/or AP2 domains was also confirmed by Pfam and SMART. The identified *CsRAVs* were named *CsRAV1* to *CsRAV8* according to the similarities of CsRAVs with their characterized counterparts in *Arabidopsis*. The information of the *CsRAVs*, including the gene ID, gene name, location, molecular weight, amino acid length and isoelectric points (pI) was listed in **Table 1**. These eight *CsRAV* genes were distributed on chromosome 1, 3, 4, 5 and 6 of cucumber, respectively (**Supplementary Figure 1**). Except for CsRAV7 and CsRAV8, the pI of the other CsRAV proteins were all greater than 7, indicating that these proteins were basic proteins (**Table 1**).

**Table 1:** Information of *RAV* genes in cucumber.

Gene ID	Gene name	Location	Molecular weight (kD)	Amino acid length (aa)	pI
*CsaV3_5G023010*	*CsRAV1*	Chr5:17061938-17063609	35.6	317	9.30
*CsaV3_5G034010*	*CsRAV2*	Chr5:27134879-27137672	38.2	344	9.18
*CsaV3_1G042340*	*CsRAV3*	Chr1:27284528-27286805	41.5	356	8.11
*CsaV3_1G028810*	*CsRAV4*	Chr1:15745012-15746031	38.8	339	8.93
*CsaV3_3G037570*	*CsRAV5*	Chr3:30990269-30994595	32.5	296	8.44
*CsaV3_6G008410*	*CsRAV6*	Chr6:6759884-6760601	21.7	184	9.93
*CsaV3_5G001410*	*CsRAV7*	Chr5:765460-770215	44.5	399	6.96
*CsaV3_4G005920*	*CsRAV8*	Chr4:3907865-3912965	43.9	384	6.40

### Phylogenetic analysis, conserved motif and gene structure analysis of *RAV* gene family in cucumber, tomato and *Arabidopsis*

**Figure 1:**
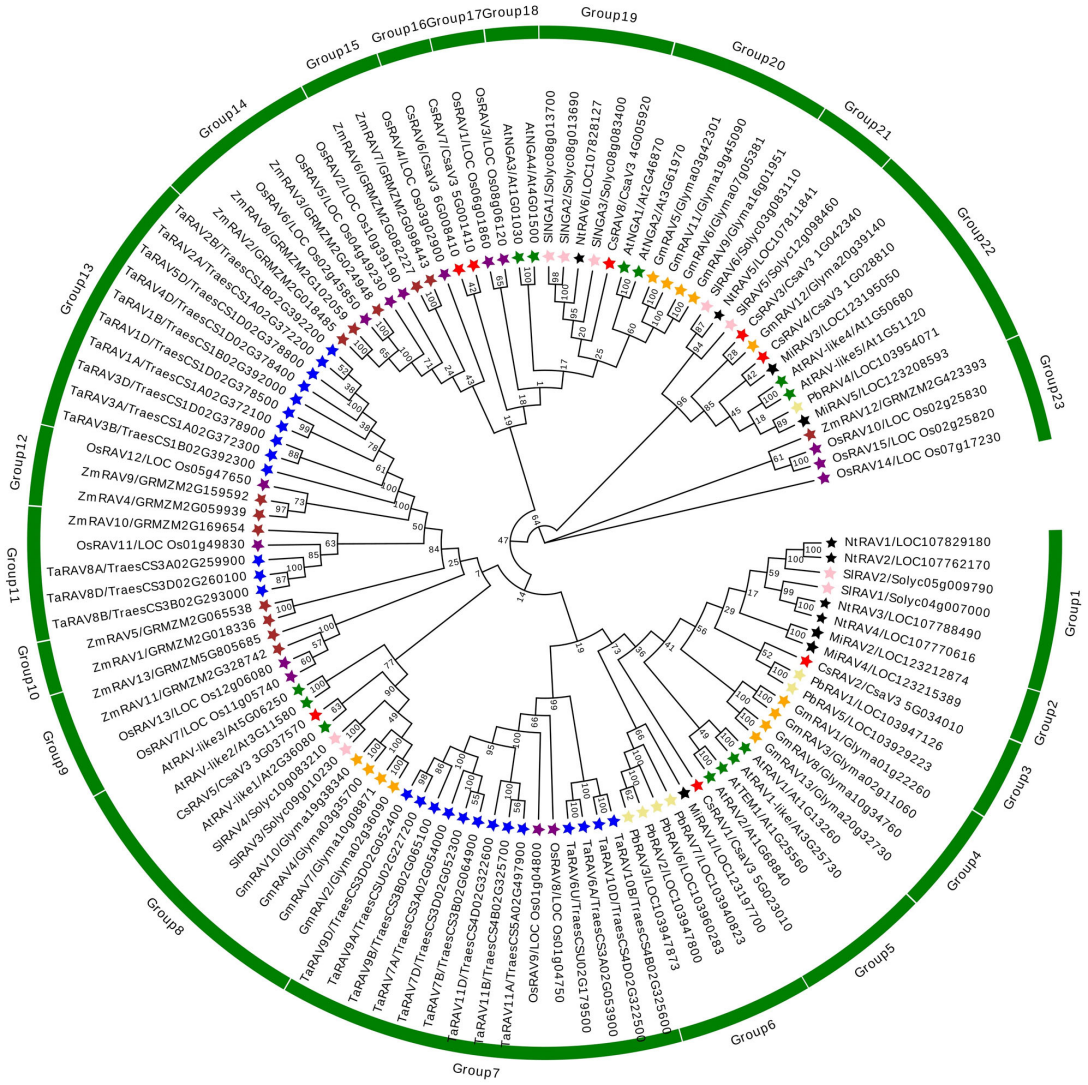
Phylogenetic relationships of 115 RAV proteins from *Cucumis sativus*, *Arabidopsis thaliana*, *Solanum lycopersicum, Glycine max, Oryza sativa, Triticum aestivum, Zea mays, Mangifera indica, Pyrus bretschneideri* and *Nicotiana tabacum*. The MEGA 7.0 software was used to construct the phylogenetic tree of 115 RAVs with complete amino acid sequences. The CsRAV proteins were marked with red stars.

**Figure 2:**
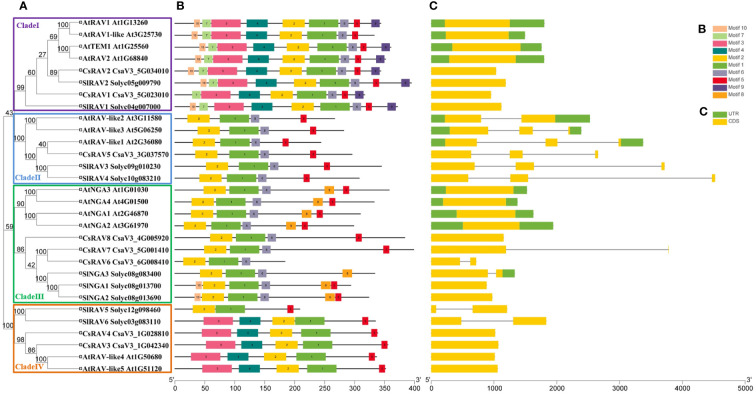
The analysis of phylogenetic relationships, conserved motifs and gene structures in *RAV* genes from cucumber, *Arabidopsis* and tomato. **(A)** A phylogenetic tree containing nine SlRAVs, 13 AtRAVs and eight CsRAVs was constructed by MEGA 7.0 software and divided into four clades named I, II, III and IV, respectively. **(B)** The conserved motifs in RAVs were identified using MEME online program. Ten conserved sequences were presented in **Supplementary Figure S2**. **(C)** Exon-intron structure of *RAV* genes.

To explore the homology of *RAV* genes among different plant species, *RAV* genes of ten species were selected to construct a phylogenetic tree based on amino acid sequences (**Figure 1**). As could be seen from **Figure 1**, the 115 RAV proteins could be roughly divided into 23 groups, among which *CsRAV* genes were classified into different groups, such as CsRAV1 was classified in group 5, CsRAV2 were classified in group3, CsRAV3 and CsRAV4 were classified in group 22, CsRAV6 and CsRAV7 were classified in group 16, and CsRAV8 was classified in group 19 (**Figure 1****;** **Supplementary Table 1**). To better assess the phylogenetic relationships of the cucumber RAV proteins, the phylogenetic tree, conserved motifs and gene structures of eight CsRAVs, 13 AtRAVs and nine SlRAVs were further analyzed in detail (**Figure 2**). As shown in **Figure 2A**, the phylogenetic tree divided these RAV proteins into four clades, which CsRAV1 and CsRAV2 were classified into Clade I, CsRAV5 belonged to Clade II, CsRAV6, CsRAV7 and CsRAV8 belonged to Clade III, and CsRAV3 and CsRAV4 were classified into Clade IV. Each clade contained *Arabidopsis*, cucumber, and tomato *RAV* genes, suggesting that proteins in the same clades might have similar functions. For example, CsRAV1 and CsRAV2 were simultaneously classified into Clade I with RAV1 and RAV2 of *Arabidopsis* and tomato, implying that CsRAV1 and CsRAV2 might regulate plant growth and improve plant tolerance (**Figure 2A**).

In order to support the phylogenetic results, the gene structure of *RAV*s from tomato, *Arabidopsis* and cucumber were analyzed (**Figure 2C**). The number of exons in *SlRAV*, *AtRAV* and *CsRAV* genes was conserved, ranging from one to three exons. As shown in **Figure 2C**, the gene structures of *RAVs* in the same clade were highly conserved in all three species. For example, all *RAVs* in Clade I contained only one exon and all *RAV*s in clade II, except *AtRAV-like2*, contained three exons. In Clade III and Clade IV, the genes had one or two exons (**Figure 2C**).

To further analyze the structural diversity and predict the function of the RAV proteins, the number and composition of conservative motifs in the SlRAVs, AtRAVs and CsRAVs were analyzed by MEME (**Figure 2B****;** **Supplementary Figure 2**). We analyzed 10 different motifs named Motif1-Motif10 (**Supplementary Figure 2**). Motifs 1 and 2, which is representative B3 domain (PF02362), were identified in all RAVs, and motifs 3 and 4, which are representative AP2 domain (PF00847), were identified in some RAV proteins, including all the RAVs in Clade I and Clade IV except SlRAV5. Some of the specific motifs were absent in specific clade (**Figure 2B**). For example, motif 6 was absent in all the member of the Clade IV subfamily, which further corroborates the accuracy of subfamily division. Motif 9 was only identified in Clade I (**Figure 2B**). Therefore, the functions of these motifs in relation to the functions of these proteins need to be investigated further.

In general, *RAV* genes with close evolutionary relationships in the phylogenetic tree contained similar conserved motifs and gene structures, suggesting that each subfamily in the three different species was evolutionarily conserved.

### Synteny analysis of *RAV* genes in cucumber, tomato and *Arabidpsis*

**Figure 3:**
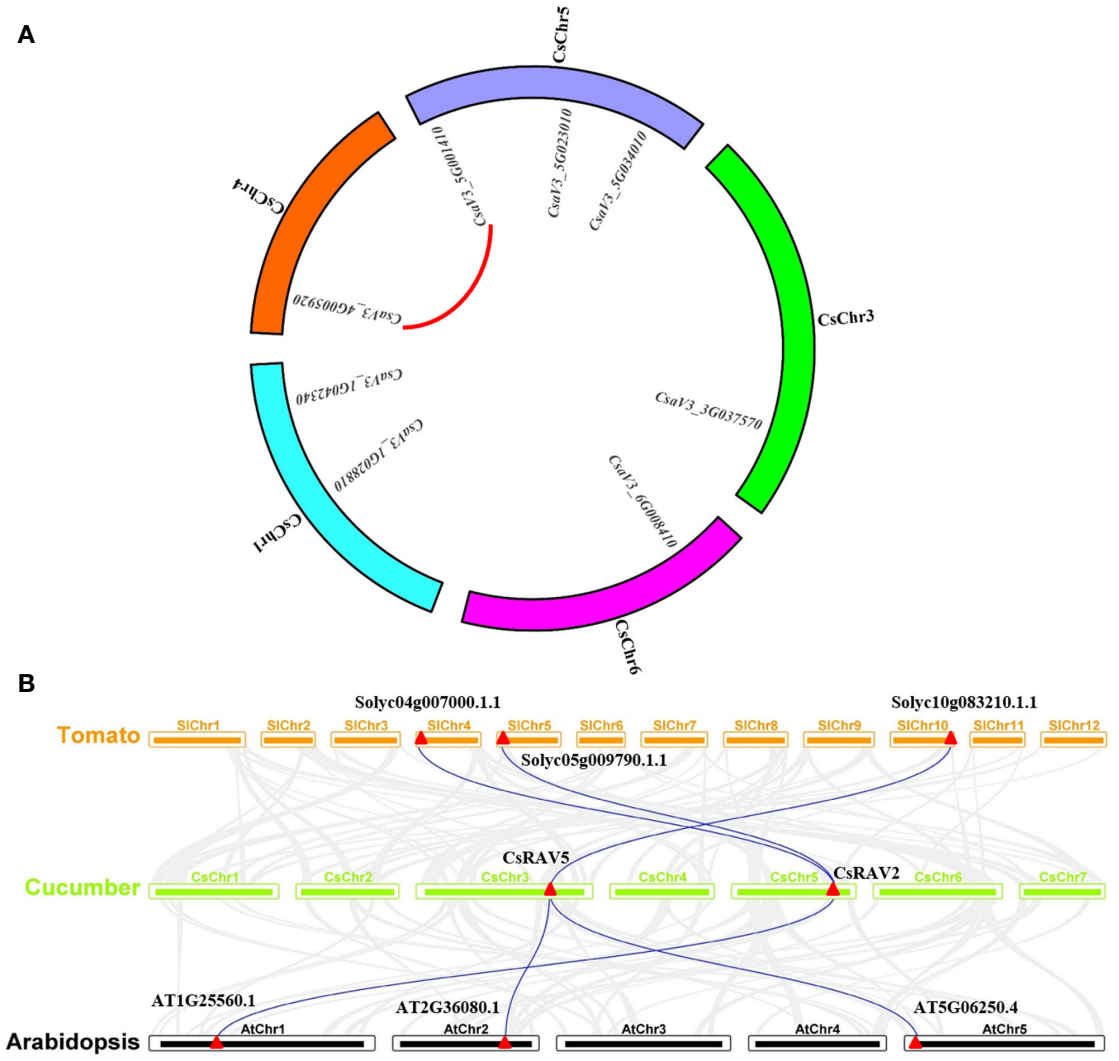
Gene duplication and synteny analysis of *CsRAV* genes. **(A)** Chromosome distribution and interchromosome relationships of *CsRAV* genes. The red line indicated segmental duplication gene pair. **(B)** Synteny analysis of *RAV* genes from cucumber, tomato and *Arabidopsis*. Gray lines represented the collinear blocks between cucumber and tomato or *Arabidopsis*; Blue lines represented the collinear blocks of *RAV* genes between cucumber and tomato or *Arabidopsis*.

Gene duplication is one of the main driving forces for the evolution of genomes and genetic systems (Das et al., 2020). Segmental duplication and tandem duplication are considered to be the two main reasons for the expansion of plant gene families (Cannon et al., 2004). To reveal the duplication of *CsRAVs*, the syntenic regions were analysed using MCscanX software. In cucumber genome, there were 231 segmental duplication blocks and 1468 tandem duplication gene pairs in all (**Supplementary Table 2**). The analysis revealed that there was one segmental duplication gene pair (*CsRAV7* and *CsRAV8*) in cucumber *RAV* gene family, but no tandem duplication gene pairs (**Figure 3A**).

To further elucidate the phylogenetic mechanism of the cucumber *RAV* family, a comparison of the syntenic map of cucumber connected with tomato and *Arabidopsis*, was constructed (**Figure 3B****;** **Supplementary Table 3**). Interestingly, only two *CsRAV* genes in cucumber were collinear with *RAV* genes in tomato and *Arabidopsis*. As shown in **Figure 3**, *CsRAV2* gene (*CsaV3_5G034010*) showed syntenic relationship with *At1G25560.1* gene in *Arabidopsis*, *Solyc04g007000.1.1* and *Solyc05g009790.1.1* genes in tomato. Similarly, *CsRAV5* gene also corresponded to syntenic gene pairs between tomato and *Arabidopsis* (**Supplementary Table 3**). These results indicated that these orthologous pairs might already exist before the ancestral divergence. In addition, we found the rest of the *CsRAV* genes (*CsRAV1*, *CsRAV3*, *CsRAV4*, *CsRAV6*, *CsRAV7* and *CsRAV8*) were not associated with syntenic gene pairs in *Arabidopsis* or tomato, suggesting that they might be unique to cucumber during the evolutionary process.

### *Cis*-element analysis of *CsRAVs* promoter in cucumber

**Figure 4:**
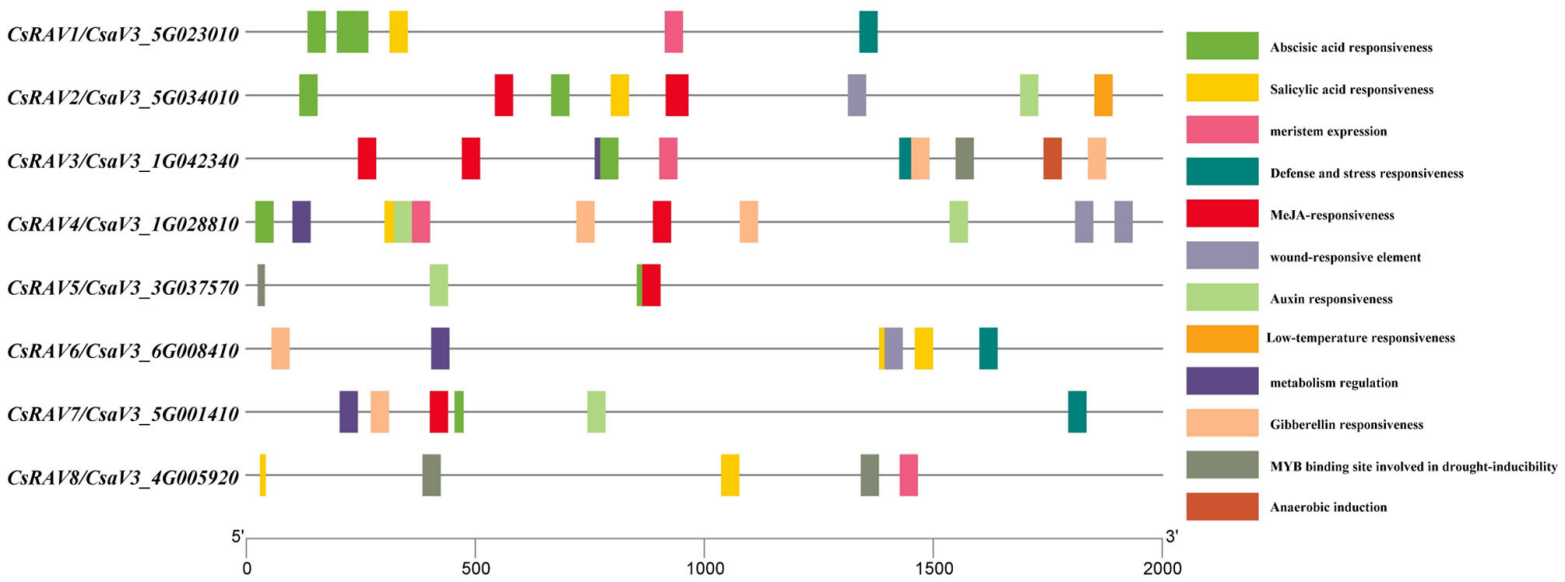
The promoter analysis of the *CsRAV* genes in cucumber. The potential *cis*-elements on the 2-kb promoter regions upstream of *CsRAV* genes were analysed using PlantCARE. Different colored boxes represented the corresponding *cis*-elements.

*Cis*-acting elements on promoters are non-coding DNA sequences that play key roles in gene expression and mutation. To explore the potential function of *CsRAV* genes in cucumber, the putative *cis*-elements on the 2-kb promoter regions of *CsRAV* were analyzed by PlantCARE (**Supplementary Table 4**). As shown in **Figure 4**, the *cis*-elements associated with abiotic stress such as low temperature, drought, wound, defense and stress existed on the promoter of *CsRAV* genes. Moreover, *cis*-elements that respond to plant hormones were also found on the promoter regions of *CsRAV* genes, such as auxin, gibberellin (GA), abscisic acid (ABA), salicylic acid (SA) and jasmonic acid (MeJA). In addition, some *cis*-elements involved in regulating plant growth and development were present on promoters of certain *CsRAV* genes, for example, meristem expression, metabolism regulation and anaerobic induction (**Figure 4**). Furthermore, the core elements including transcription start site (TSS) and TATA-box of eight *CsRAV* genes were predicted using TSSPlant online software. The results showed that only four transcription start sites were predicted for *CsRAV3* and no TATA-box, while three core elements were predicted for *CsRAV5* and *CsRAV7* and two core elements were predicted for all the other five genes (**Supplementary Figure 3**). In conclusion, the analyses of *cis*-elements suggested that *CsRAVs* might play vital functions in abiotic stress responses and multiple plant hormone signaling pathways.

### Responses of *CsRAV* genes to different abiotic stresses

**Figure 5:**
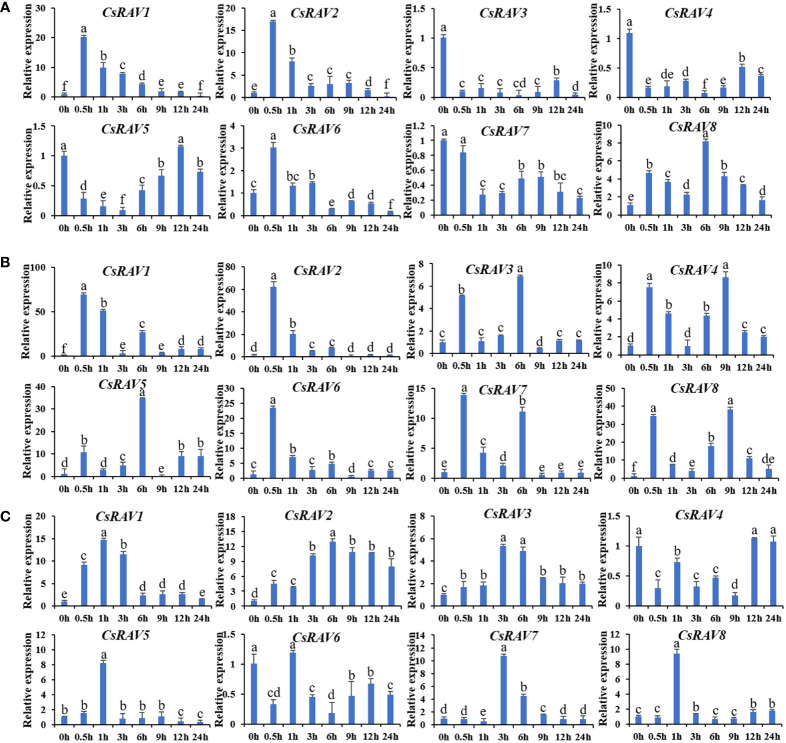
Relative expression of *CsRAV* genes in cucumber seedlings treated with NaCl, ABA and low temperature. QRT-PCR was used to analyze the expression levels of *CsRAVs* under NaCl (100 mM) **(A)**, ABA (100 μM) **(B)** and 4°C **(C)**. Using cucumber *β-actin* gene as an internal control, three biological replicates were used to analyze gene expression. Error bars were the standard errors (SE). Different lowercase letters represented significant differences (P < 0.05).

Abiotic stresses are known to affect many physiological processes. To explore the response of *CsRAV* genes to different stresses, qRT-PCR was used to analyze the expression patterns of these genes under low temperature (4°C), ABA treatment (100 μΜ ABA) and salt stress (100 mM NaCl), respectively (**Figure 5**). Under salt stress, *CsRAV1* and *CsRAV2* genes were significantly up-regulated (>15-fold) after 0.5 h. Two genes, *CsRAV6* and *CsRAV8*, were slightly upregulated, while others were obviously down-regulated (**Figure 5A**). Under ABA treatment, the expression levels of all *CsRAV* genes were markedly induced (>7-fold). For example, the expression levels of *CsRAV5* and *CsRAV8* were more than 30 times higher. In particular, the expression patterns of *CsRAV1* and *CsRAV2* genes showed a similar trend and the expression levels of these two genes were up-regulated 60 times after 0.5 h (**Figure 5B**). After low temperature treatment, all *CsRAV* genes were up-regulated except *CsRAV4* and *CsRAV6*, and *CsRAV1* and *CsRAV2* were up-regulated most significantly. *CsRAV1* reached maximum values at 1 h, while the expression levels of *CsRAV2* reached its maximum at 6 h (**Figure 5C**). These results suggested that some *CsRAV* genes were involved in low temperature, salt and ABA responses.

### Subcellular localization of cucumber RAVs

**Figure 6:**
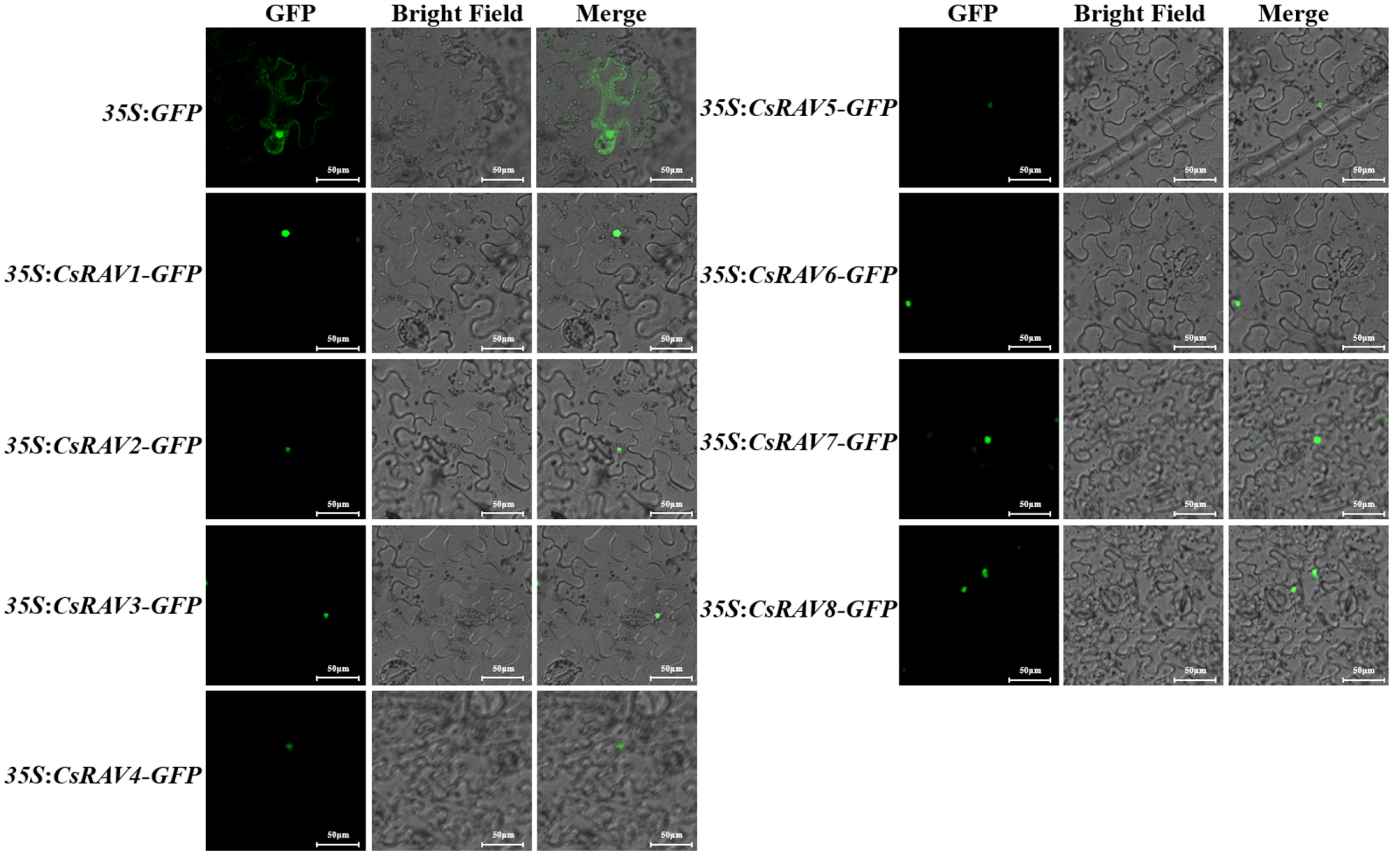
Subcellular localization of the cucumber RAV proteins in tobacco leaf. The green fluorescence signal was observed under confocal microscope 48 h after transformation.

To confirm the subcellular localization of CsRAV proteins, the GFP fusion protein vectors *35S*:*CsRAVs*-*GFP* of eight *CsRAV* genes were constructed, respectively. The *35S*:*GFP* vector was used as empty control. The green fluorescent signal of *GFP* expression in epidermal cells of tobacco leaves was observed after transformation. Under confocal laser scanning microscope, the different CsRAV fusion proteins were all localized in the nucleus, while *35S*:*GFP* fluorescence signal was distributed throughout the whole cell (**Figure 6**). The data showed that all CsRAV proteins were nuclear localization proteins, suggesting that these CsRAVs might be involved in transcriptional regulation.

### Overexpression of *CsRAV1* improved cucumber and transgenic *Arabidopsis* tolerance to NaCl and ABA

**Figure 7:**
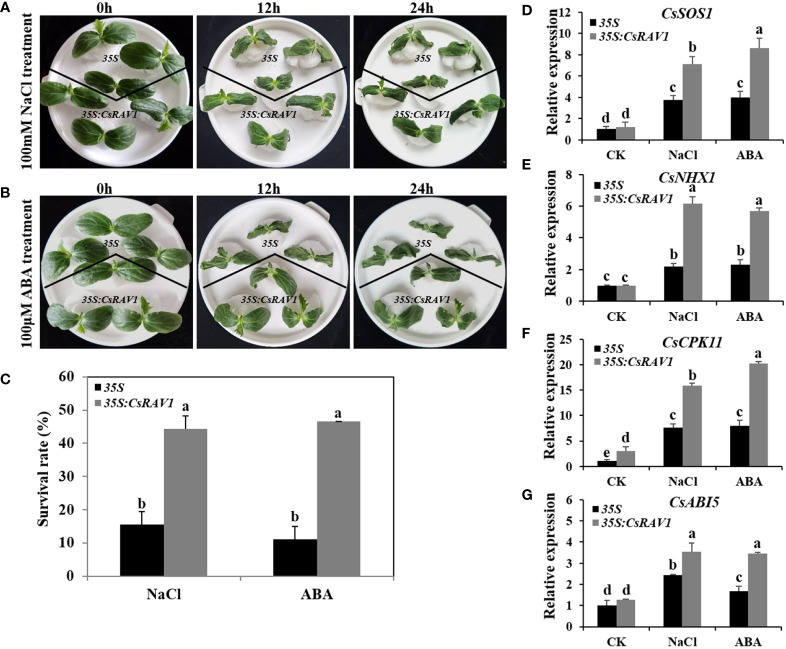
Overexpression of *CsRAV1* improved NaCl and ABA tolerance in cucumber seedlings. **(A, B)** Phenotypes of *35S* and *35S*:*CsRAV1* cucumber seedlings treated with NaCl and ABA under hydroponics. **(C)** Survival rate of *35S* and *35S*:*CsRAV1* plants after 24 h NaCl and ABA treatments. The transcript levels of *CsSOS1* **(D)**, *CsNHX1* **(E)**, *CsCPK11* **(F)**, and *CsABI5* **(G)** genes in *35S* and *35S*:*CsRAV1* cucumber seedlings were analyzed with qRT-PCR under NaCl and ABA treatment for 6 h. The cucumber *β-actin* gene was used as internal control. Error bars were the standard errors (SE). Different letters indicated significant differences (P < 0.05).

The expression analysis showed that *CsRAV1* was significantly induced by NaCl and ABA in cucumber (**Figures 5A, B****)**. To determine the function of *CsRAV1* in abiotic stress response, first, *agrobacterium*-mediated transient transformation of cucumber cotyledons was used to elucidate the tolerance of *CsRAV1* to NaCl and ABA. In the preliminary experiments, the transient transfection of *CsRAV1* was successfully verified by PCR amplification, and the expression levels of *CsRAV1* in transgenic cucumber cotyledons were observably higher than that in *35S* plants by qRT-PCR (**Supplementary Figure 4**). When cultured in a hydroponic solution containing 100 mM NaCl, the control seedlings (overexpressing *35S* empty vector) showed significant wilting after 12 h compared with overexpressed *CsRAV1* transgenic seedlings, and the difference in wilting degree was more obvious after 24 h (**Figure 7A**). After 24 h of NaCl treatment, the survival rate of transgenic seedlings was 44%, which was significantly higher than that of control, indicating that the overexpression of *CsRAV1* produced significant salt tolerance (**Figure 7C**). Similarly, in the presence of exogenous ABA, the growth of both control and *35S*:*CsRAV1* seedlings was distinctly harmed, but the damage of control was much greater than that of transgenic seedlings. After 24 h of ABA treatment, about 46% of the transgenic seedlings remained green with extended cotyledons, while 89% of the control showed obvious injury symptoms, such as wilting and death (**Figures 7B, C****)**. To elucidate the underlying regulatory effect of *CsRAV1* on tolerance to NaCl and ABA stress, the expression levels of four stress- and ABA- related marker genes were detected by qRT-PCR in *35S* and *35S*:*CsRAV1* cucumber seedlings before and after NaCl and ABA treatments. As shown in **Figures 7D–G**, only *CsCPK11* was slightly higher expressed in *35S*:*CsRAV1* transgenic plants before NaCl and ABA treatments. However, after NaCl and ABA treatments, all the four genes, *CsSOS1* (Salt overly Sensitive 1), *CsNHX1* (vacuolar sodium/proton antiporter), *CsCPK11* (calcium-dependent protein kinase) and *CsABI5* (ABA-insensitive factor 5) were strongly induced in *35S* and *35S*:*CsRAV1* plants. Furthermore, the expression levels of the four genes were significantly higher in *35S*:*CsRAV1* transgenic plants than those in *35S* plants after NaCl and ABA stress treatment. The enhanced expression of these genes in *35S*:*CsRAV1* transgenic plants might conduce to improve plant stress resistance, which also suggested that *CsRAV1* may be involved in response to NaCl and ABA stress by influencing the transcription of multiple stress-related genes.

**Figure 8:**
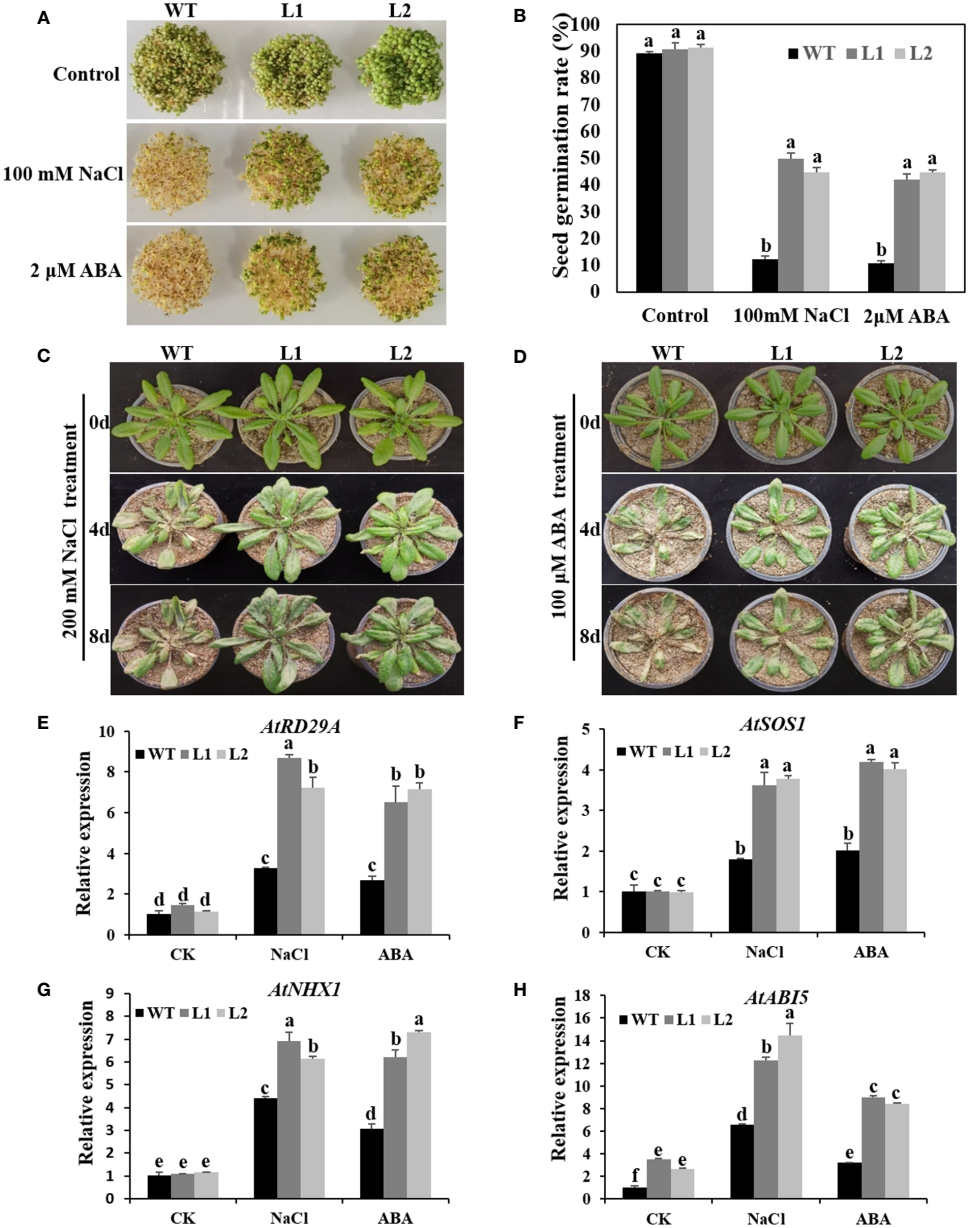
*CsRAV1* enhanced NaCl and ABA tolerance in transgenic *Arabidopsis*. **(A)** Seed germination of WT and *35S*:*CsRAV1* plants (L1 and L2) after 7 d on 1/2 MS with NaCl and ABA. **(B)** Seed germination rate in **(A)**. Different letters indicated significant differences (P < 0.05). **(C, D)** Phenotypes of WT and *35S*:*CsRAV1* transgenic plants treated with NaCl and ABA. After 6 h of NaCl and ABA stress treatment, the expression levels of stress- and ABA- related marker genes *AtRD29A* **(E)**, *AtSOS1* **(F)**, *AtNHX1* **(G)**, and *AtABI5* **(H)** were detected in WT and *35S*:*CsRAV1* plants (L1 and L2). The *actin* gene was used as internal control, with error bars of three biological replicates. Different letters indicated significant differences (P < 0.05).

To further study the function of *CsRAV1* in plant resistance to abiotic stress, we obtained transgenic *Arabidopsis* plants overexpressing *CsRAV1* and two highly expressed homozygous transgenic lines (L1 and L2) were selected for further analysis (**Supplementary Figure 5**). The NaCl and ABA tolerance of *CsRAV1* transgenic plants was evaluated. For germination assays, the WT, L1 and L2 on 1/2 MS medium showed similar germination rate (**Figure 8A**). However, the germination of both WT and *35S*:*CsRAV1* transgenic seeds was significantly inhibited on 1/2 MS medium containing 100 mM NaCl or 2 μM ABA, but the inhibition of the WT was much higher than that of the transgenic seeds. With 100 mM NaCl or 2 μM ABA treatment, nearly 40-50% of *CsRAV1* transgenic seeds were able to germinate, while only about 10-12% of the WT seeds could germinate (**Figures 8A, B****)**. The results showed that overexpressing *CsRAV1* could remarkably improve seed germination rate under salt and ABA treatment. In addition, we determined the seedling growth of 3-week-old WT and *CsRAV1* transgenic lines under 200 mM NaCl and 100μM ABA treatment, respectively. After four days of treatment with 200 mM NaCl or 100μM ABA, the leaves of *CsRAV1* transgenic lines were still green, but the leaves of WT were severely yellowed (**Figures 8C, D****)**. After eight days, the *CsRAV1* transgenic lines and WT had more obvious differences in NaCl and ABA resistance, indicating that *CsRAV1* transgenic plants were more tolerant to NaCl and ABA stresses than WT. Following NaCl and ABA stress treatment, the expression levels of stress- and ABA- related marker genes were significantly up-regulated in transgenic lines (L1 and L2) and WT, but the induction levels of *AtRD29A* (response to desiccation 29A), *AtSOS1*, *AtNHX1* and *AtABI5* genes were markedly higher in L1 and L2 lines than in WT (**Figures 8E–H**). In conclusion, these results indicated that *CsRAV1* might play key roles in ABA signaling and in plant response to high salinity during seed germination and seedling development.

## Discussion

*RAV* gene family is widely distributed in higher plants and is one of the plant-specific regulatory gene families. As one of the *B3* gene superfamilies in plants, the *RAV* family genes regulate many aspects of plant growth and development, including regulating flowering time and heading date (Hu et al., 2004; Woo et al., 2010; Duan et al., 2016; Shin and Nam, 2018). At present, *RAV* genes have been extensively studied in *Arabidopsis*, rice, soybean, cotton, pepper and other plants (Giraudat et al., 1992; Sohn et al., 2006; Zhao et al., 2008; Li et al., 2015; Osnato et al., 2020). However, little is known about the role of the *RAVs* in cucumber. In this study, we identified eight cucumber *RAV* genes through genome-wide analysis. The phylogenetic relationship, gene structure, conserved domains, gene duplication events and *cis*-acting elements on promoters of *RAV* family genes in cucumber were systematically analyzed.

To analyze the molecular evolutionary relationship between cucumber RAV proteins and RAVs among other plant species, a phylogenetic tree of 115 RAV proteins from ten species was constructed, and roughly divided into 23 groups, among which CsRAVs were classified into different groups (**Figure 1**). To further study the evolutionary relationship and diversity/conservativeness of *RAV* genes in tomato, *Arabidopsis* and cucumber, the phylogenetic tree, conserved motifs and gene structures of eight CsRAVs, 13 AtRAVs and nine SlRAVs were further analyzed in detail (**Figure 2**). The gene structure pattern and motif composition can give important insights for evolutionary relationships of multi-gene families (Boudet et al., 2001; Babenko et al., 2004). Tomato, *Arabidopsis* and cucumber are different in anatomy and physiology. Therefore, some branches may have different ways of expansion in the *RAV* family of tomato, *Arabidopsis* and cucumber. As shown in **Figure 2**, the *RAVs* within the same clade shared similar gene structure and motif composition. Genes with similar gene structures and conserved motifs usually have similar functions. The cucumber RAV proteins were clustered into some *Arabidopsis* functional clades, which will provide valuable information for studying the function of *CsRAV* genes.

Previous studies have investigated the role of RAV transcription factors in regulating plant responses to biotic and abiotic stresses such as plant hormones and pathogens (Chen et al., 2021). In our study, it was shown that the promoters of *CsRAV* genes contain *cis*-elements that respond to abiotic stresses and hormones such as drought, wound, defense and stress, low temperature, auxin, salicylic acid, abscisic acid, gibberellin and jasmonic acid (**Figure 4**). In addition, TATA box is one of the elements that constitute the eukaryotic promoters, and its sequence is TATA (A/T) A (A/T). It is generally about -30bp (-25~-32bp) upstream of the transcription start site of most eukaryotic genes. The TATA box is the selection that determines the initiation of gene transcription and is one of the binding sites for RNA polymerase, which can only be transcribed after the RNA polymerase is firmly bound to the TATA box (Pedersen et al., 1999). The transcription start site (TSS) is the location where transcription starts at the 5’-end of a gene sequence (Peng et al., 2006). Our data showed that all *CsRAV* gene promoters except *CsRAV3* contained TSS and TATA-box (**Supplementary Figure 3**). The specific TSS and TATA-box on each *CsRAV* gene promoter need to be determined by robust analysis of 5’-transcript ends (5’-RATE) experiment. Previous studies indicated that *AtRAV1*, *AtRAV2*, *BnaRAV-1*, *GhRAV1* and *CARAV1* genes could be significantly induced after stress treatment (Sohn et al., 2006; Zhuang et al., 2011; Fu et al., 2014; Li et al., 2015). In this study, *CsRAV* genes were mainly up-regulated by salt, low temperature and ABA, and some of them were significantly affected in different degrees (**Figure 5**). These results suggested that the cucumber *RAV* genes, like the genes of other plants, played key roles in stress resistance. However, there were different response patterns under different stresses, indicating functional diversity among the genes.

The functions of *RAV* genes in *Arabidopsis*, soybean, cotton, pepper and rice have been widely reported (Hu et al., 2004; Zhao et al., 2017; Chen et al., 2021; Kabir et al., 2021). Overexpression of *AtRAV1* and *AtRAV2* can improve the drought resistance in cotton (Matías-Hernández et al., 2014). Overexpression of soybean *GmRAV3* in *Arabidopsis* could significantly increase the resistance of transgenic lines to high salt and drought, and lead to the insensitivity of transgenic plants to exogenous ABA (Lu et al., 2014; Zhao et al., 2017). *CsRAV1* was significantly induced by salt and ABA (**Figures 5A, B****)**, so *CsRAV1* might play key roles in resistance to salt or ABA stress. To further determine the roles of *CsRAV1* in abiotic stress response, we obtained the *35S*:*CsRAV1* transgenic *Arabidopsis* and transient transformed cucumber cotyledons. Under NaCl and ABA treatments, we found *35S*:*CsRAV1* cucumber seedlings indeed showed enhanced tolerance to salt and ABA compared with *35S* plants (**Figures 7A–C**). Meanwhile, the transgenic *Arabidopsis* showed higher germination rates than WT, indicating that *CsRAV1* played vital roles in seed germination and development (**Figures 8A–D**). The expression of *CsRAV1* was also induced by low temperature (**Figure 5C**), but we found that there were no significant differences in phenotype between *35S*:*CsRAV1* transgenic seedlings and control treated at 4°C, which required further verification and discussion in the future.

Previous studies have shown that abiotic stress-related marker genes (such as *RD29A*, *ABI5*, *SOS1*, *SOS2*, *NHX3*, *CPK1*, *RD22* and *APX2*) are involved in plant response and defense against environmental stress (Li et al., 2015; Zhang et al., 2020). Changes in the expression of these marker genes may contribute to plant resistance to abiotic stress. The transcriptional levels of stress- and ABA- related genes were regulated in transgenic plants under different stresses (Li et al., 2015; Zhang et al., 2020). Under NaCl treatment, the expressions of *ABI1*, *RD29A* and *RAB18* were obvious enhanced in *35S*:*GhRAV1* transgenic plants compared with those in WT (Li et al., 2015). Compared with those in WT, the transcriptional levels *AtSOS1*, *AtRD22* and *AtRD29A* were upregulated in *35S*:*TaRAV1* transgenic plants after NaCl treatment (Luo et al., 2022). The *ABI5* gene is a key positive regulator of ABA signal transduction, and increased *ABI5* gene expression enhances susceptibility to ABA during seed germination and early seedling development (Lopez-Molina et al., 2001). AtRAV1 is involved in ABA signaling by directly binding to the promoter of *ABI5* and influencing its expression (Feng et al., 2014). MdRAV1 plays a key role in ABA signaling by directly binding to the promoters of *MdABI3* and *MdABI4* (Zhao et al., 2019b). Previous research showed the sodium/proton antiporter1 (NHX1) maintained Na^+^ homeostasis in plants and plays a vital role in plant salt tolerance (Zhang et al., 2020). In this study, qRT-PCR analysis indicated that the transcriptional levels of four stress-related genes, including *CsSOS1*, *CsNHX1*, *CsCPK11* and *CsABI5*, in transgenic cucumber were higher than those in *35S* plants after NaCl and ABA treatment (**Figures 7D–G**). These results indicated that CsRAV1 might directly regulate the expression of *CsABI5*, thereby participating in ABA signal pathway and enhancing ABA tolerance in cucumber. Furthermore, CsRAV1 might regulate the transcription of *CsSOS1* and *CsNHX1* genes to maintain ion homeostasis, thus enhancing salt tolerance. In addition, stress- and ABA- related marker genes (*AtRD29A*, *AtSOS1*, *AtNHX1* and *AtABI5*) in transgenic *Arabidopsis* were significantly up-regulated compared with WT, suggesting that *CsRAV1* might alleviate NaCl and ABA stress by regulating stress- related genes in *Arabidopsis* **(** **Figures 8E–H****)**. These results will provide a theoretical basis for further study on the function of RAV transcription factor in cucumber.

## Conclusions

In this study, the cucumber *RAV* family genes were systematically analyzed. The expression patterns of *CsRAV* genes under different stress treatments were studied, and the potential functions of *CsRAV1* were also analyzed using the transgenic method. This work provides a rich insight into the functions and regulatory mechanism of *CsRAV* genes in abiotic stress resistance of cucumber.

## Data availability statement

The original contributions presented in the study are included in the article/**Supplementary Material**. Further inquiries can be directed to the corresponding authors.

## Author contributions

JL, WH and ZW conceived and designed the experiments. JL, CS, HL, SW, LH and YY performed the experiments. JL analyzed the data and wrote the manuscript. WH and ZW revised the manuscript. All authors contributed to the article and approved the submitted version.

## References

[B1] Alonso J. M. Stepanova A. N. Leisse T. J. Kim C. J. Chen H. Shinn P.. (2003). Genome-wide insertional mutagenesis of *Arabidopsis thaliana* . Science 301 (5633), 653–657. doi: 10.1126/science.1086391 12893945

[B2] Alvarez J. P. Goldshmidt A. Efroni I. Bowman J. L. Eshed Y. (2009). The NGATHA distal organ development genes are essential for style specification in *Arabidopsis* . Plant Cell 21 (5), 1373–1393. doi: 10.1105/tpc.109.065482 19435933 PMC2700527

[B3] Babenko V. N. Rogozin I. B. Mekhedov S. L. Koonin E. V. (2004). Prevalence of intron gain over intron loss in the evolution of paralogous gene families. Nucleic Acids Res. 32 (12), 3724–3733. doi: 10.1093/nar/gkh686 15254274 PMC484173

[B4] Boudet N. Aubourg S. Toffano-Nioche C. Kreis M. Lecharny A. (2001). Evolution of intron/exon structure of DEAD helicase family genes in *Arabidopsis*, *Caenorhabditis*, and *Drosophila* . Genome Res. 11 (12), 2101–2114. doi: 10.1101/gr.200801 11731501 PMC311229

[B5] Cannon S. B. Mitra A. Baumgarten A. Young N. D. May G. (2004). The roles of segmental and tandem gene duplication in the evolution of large gene families in *Arabidopsis* thaliana. BMC Plant Biol. 4, 10. doi: 10.1186/1471-2229-4-10 15171794 PMC446195

[B6] Castillejo C. Pelaz S. (2008). The balance between CONSTANS and TEMPRANILLO activities determines FT expression to trigger flowering. Curr. Biol. 18 (17), 1338–1343. doi: 10.1016/j.cub.2008.07.075 18718758

[B7] Chen C. Chen H. Zhang Y. Thomas H. R. Frank M. H. He Y.. (2020). TBtools: An integrative toolkit developed for interactive analyses of big biological data. Mol. Plant 13 (8), 1194–1202. doi: 10.1016/j.molp.2020.06.009 32585190

[B8] Chen C. Li Y. Zhang H. Ma Q. Wei Z. Chen J.. (2021). Genome-wide analysis of the *RAV* transcription factor genes in rice reveals their response patterns to hormones and virus infection. Viruses. 13 (5), 752. doi: 10.3390/v13050752 33922971 PMC8146320

[B9] Das L. S. Dutta S. Schäffner A. R. Das M. (2020). Gene duplication and stress genomics in brassicas: Current understanding and future prospects. J. Plant Physiol. 255, 153293. doi: 10.1016/j.jplph.2020.153293 33181457

[B10] Duan Y. B. Li J. Qin R. Y. Xu R. F. Li H. Yang Y. C.. (2016). Identification of a regulatory element responsible for salt induction of rice *OsRAV2* through ex situ and *in situ* promoter analysis. Plant Mol. Biol. 90 (1-2), 49–62. doi: 10.1007/s11103-015-0393-z 26482477

[B11] Feng C. Z. Chen Y. Wang C. Kong Y. H. Wu W. H. Chen Y. F. (2014). *Arabidopsis* RAV1 transcription factor, phosphorylated by SnRK2 kinases, regulates the expression of *ABI3*, *ABI4*, and *ABI5* during seed germination and early seedling development. Plant J. 80 (4), 654–668. doi: 10.1111/tpj.12670 25231920

[B12] Fu M. Kang H. K. Son S. H. Kim S. K. Nam K. H. (2014). A subset of *Arabidopsis* RAV transcription factors modulates drought and salt stress responses independent of ABA. Plant Cell Physiol. 55 (11), 1892–1904. doi: 10.1093/pcp/pcu118 25189341

[B13] Giraudat J. Hauge B. M. Valon C. Smalle J. Parcy F. Goodman H. M. (1992). Isolation of the *Arabidopsis ABI3* gene by positional cloning. Plant Cell 4 (10), 1251–1261. doi: 10.1105/tpc.4.10.1251 1359917 PMC160212

[B14] Hu Y. X. Wang Y. X. Liu X. F. Li J. Y. (2004). *Arabidopsis RAV1* is down-regulated by brassinosteroid and may act as a negative regulator during plant development. Cell Res. 14 (1), 8–15. doi: 10.1038/sj.cr.7290197 15040885

[B15] Huang S. Li R. Zhang Z. Li L. Gu X. Fan W.. (2009). The genome of the cucumber, *Cucumis sativus* l. Nat. Genet. 41 (12), 1275–1281. doi: 10.1038/ng.475 19881527

[B16] Kabir N. Lin H. Kong X. Liu L. Qanmber G. Wang Y.. (2021). Identification, evolutionary analysis and functional diversification of *RAV* gene family in cotton (G. hirsutum l.). Planta. 255 (1), 14. doi: 10.1007/s00425-021-03782-2 34862931

[B17] Kagaya Y. Ohmiya K. Hattori T. (1999). RAV1, a novel DNA-binding protein, binds to bipartite recognition sequence through two distinct DNA-binding domains uniquely found in higher plants. Nucleic Acids Res. 27, 470–478. doi: 10.1093/nar/27.2.470 9862967 PMC148202

[B18] Krzywinski M. Schein J. Birol I. Connors J. Gascoyne R. Horsman D.. (2009). Circos: an information aesthetic for comparative genomics. Genome Res. 19 (9), 1639–1645. doi: 10.1101/gr.092759.109 19541911 PMC2752132

[B19] Kwon S. H. Lee B. H. Kim E. Y. Seo Y. S. Lee S. Kim W. T.. (2009). Overexpression of a *Brassica rapa NGATHA* gene in *Arabidopsis thaliana* negatively affects cell proliferation during lateral organ and root growth. Plant Cell Physiol. 50 (12), 2162–2173. doi: 10.1093/pcp/pcp150 19880400

[B20] Lee B. H. Kwon S. H. Lee S. J. Park S. K. Song J. T. Lee S.. (2015). The *Arabidopsis thaliana* NGATHA transcription factors negatively regulate cell proliferation of lateral organs. Plant Mol. Biol. 89 (4-5), 529–538. doi: 10.1007/s11103-015-0386-y 26433582

[B21] Li C. W. Su R. C. Cheng C. P. Sanjaya You S. J. Hsieh T. H.. (2011). Tomato RAV transcription factor is a pivotal modulator involved in the AP2/EREBP-mediated defense pathway. Plant Physiol. 156 (1), 213–227. doi: 10.1104/pp.111.174268 21398258 PMC3091068

[B22] Li J. Li H. Quan X. Shan Q. Wang W. Yin N.. (2022). Comprehensive analysis of cucumber c-repeat/dehydration-responsive element binding factor family genes and their potential roles in cold tolerance of cucumber. BMC Plant Biol. 22 (1), 270. doi: 10.1186/s12870-022-03664-z 35655135 PMC9161515

[B23] Li J. Wang T. Han J. Ren Z. (2020). Genome-wide identification and characterization of cucumber bHLH family genes and the functional characterization of *CsbHLH041* in NaCl and ABA tolerance in *Arabidopsis* and cucumber. BMC Plant Biol. 20 (1), 272. doi: 10.1186/s12870-020-02440-1 32527214 PMC7291561

[B24] Li X. J. Li M. Zhou Y. Hu S. Hu R. Chen Y.. (2015). Overexpression of cotton *RAV1* gene in *Arabidopsis* confers transgenic plants high salinity and drought sensitivity. PLoS One 10 (2), e0118056. doi: 10.1371/journal.pone.0118056 25710493 PMC4340050

[B25] Lopez-Molina L. Mongrand S. Chua N. H. (2001). A postgermination developmental arrest checkpoint is mediated by abscisic acid and requires the ABI5 transcription factor in *Arabidopsis.* Proc. Natl. Acad. Sci. U.S.A. 98, 8, 4782–4787. doi: 10.1073/pnas.081594298 11287670 PMC31911

[B26] Lu Q. Zhao L. Li D. Hao D. Zhan Y. Li W. (2014). A GmRAV ortholog is involved in photoperiod and sucrose control of flowering time in soybean. PLoS One 9 (2), e89145. doi: 10.1371/journal.pone.0089145 24551235 PMC3925180

[B27] Luo Y. X. Chen S. K. Wang P. D. Peng D. Zhang X. Li H. F.. (2022). Genome-wide analysis of the *RAV* gene family in wheat and functional identification of *TaRAV1* in salt stress. Int. J. Mol. Sci. 23 (16), 8834. doi: 10.3390/ijms23168834 36012100 PMC9408559

[B28] Magnani E. Sjölander K. Hake S. (2004). From endonucleases to transcription factors: evolution of the AP2 DNA binding domain in plants. Plant Cell. 16 (9), 2265–2277. doi: 10.1105/tpc.104.023135 15319480 PMC520932

[B29] Marín-González E. Matías-Hernández L. Aguilar-Jaramillo A. E. Lee J. H. Ahn J. H. Suárez-López P.. (2015). SHORT VEGETATIVE PHASE up-regulates *TEMPRANILLO2* floral repressor at low ambient temperatures. Plant Physiol. 169 (2), 1214–1224. doi: 10.1104/pp.15.00570 26243615 PMC4587448

[B30] Matías-Hernández L. Aguilar-Jaramillo A. E. Marín-González E. Suárez-López P. Pelaz S. (2014). *RAV* genes: regulation of floral induction and beyond. Ann. Botany. 114 (7), 1459–1470. doi: 10.1093/aob/mcu069 24812253 PMC4204781

[B31] Osnato M. Matias-Hernandez L. Aguilar-Jaramillo A. E. Kater M. M. Pelaz S. (2020). Genes of the *RAV* family control heading date and carpel development in rice. Plant Physiol. 183 (4), 1663–1680. doi: 10.1104/pp.20.00562 32554473 PMC7401134

[B32] Pedersen A. G. Baldi P. Chauvin Y. Brunak S. (1999). The biology of eukaryotic promoter prediction–a review. Comput. Chem. 23, 191–207. doi: 10.1016/S0097-8485(99)00015-7 10404615

[B33] Peng Z. H. Chen J. Cao L. J. Gao T. T. (2006). Identification of TSS in the human genome based on a RBF neural network. Int. J. Automation Computing. 3 (1), 35–40. doi: 10.1007/s11633-006-0035-7

[B34] Romanel E. A. Schrago C. G. Couñago R. M. Russo C. A. Alves-Ferreira M. (2009). Evolution of the B3 DNA binding superfamily: new insights into *REM* family gene diversification. PLoS One 4 (6), e5791. doi: 10.1371/journal.pone.0005791 19503786 PMC2688026

[B35] Shin H. Y. Nam K. H. (2018). RAV1 negatively regulates seed development by directly repressing *MINI3* and *IKU2* in *Arabidopsis* . Molecules Cells 41 (12), 1072–1080. doi: 10.14348/molcells.2018.0259 30518173 PMC6315318

[B36] Sohn K. H. Lee S. C. Jung H. W. Hong J. K. Hwang B. K. (2006). Expression and functional roles of the pepper pathogen-induced transcription factor RAV1 in bacterial disease resistance, and drought and salt stress tolerance. Plant Mol. Biol. 61 (6), 897–915. doi: 10.1007/s11103-006-0057-0 16927203

[B37] Swaminathan K. Peterson K. Jack T. (2008). The plant *B3* superfamily. Trends Plant Sci. 13 (12), 647–655. doi: 10.1016/j.tplants.2008.09.006 18986826

[B38] Wang Y. Deng D. Zhang R. Wang S. Bian Y. Yin Z. (2012a). Systematic analysis of plant-specific B3 domain-containing proteins based on the genome resources of 11 sequenced species. Mol. Biol. Rep. 39 (5), 6267–6282. doi: 10.1007/s11033-012-1448-8 22302388

[B39] Wang Y. Tang H. Debarry J. D. Tan X. Li J. Wang X.. (2012b). MCScanX: a toolkit for detection and evolutionary analysis of gene synteny and collinearity. Nucleic Acids Res. 40 (7), e49. doi: 10.1093/nar/gkr1293 22217600 PMC3326336

[B40] Wei Y. Chang Y. Zeng H. Liu G. He C. Shi H. (2018). RAV transcription factors are essential for disease resistance against cassava bacterial blight *via* activation of melatonin biosynthesis genes. J. Pineal Res. 64 (1). doi: 10.1111/jpi.12454 29151275

[B41] Woo H. R. Kim J. H. Kim J. Kim J. Lee U. Song I. J.. (2010). The RAV1 transcription factor positively regulates leaf senescence in *Arabidopsis* . J. Exp. Bot. 61 (14), 3947–3957. doi: 10.1093/jxb/erq206 20826506 PMC2935868

[B42] Zhang X. Chen L. Shi Q. Ren Z. (2020). *SlMYB102*, an R2R3-type MYB gene, confers salt tolerance in transgenic tomato. Plant Sci. 291, 110356. doi: 10.1016/j.plantsci.2019.110356 31928668

[B43] Zhao L. Luo Q. Yang C. Han Y. Li W. (2008). A RAV-like transcription factor controls photosynthesis and senescence in soybean. Planta 227 (6), 1389–1399. doi: 10.1007/s00425-008-0711-7 18297307

[B44] Zhao L. Zhang F. Liu B. Yang S. Xiong X. Hassani D.. (2019a). *CmRAV1* shows differential expression in two melon (*Cucumis melo* l.) cultivars and enhances salt tolerance in transgenic *Arabidopsis* plants. Acta Biochim. Biophys. Sin. (Shanghai) 51 (11), 1123–1133. doi: 10.1093/abbs/gmz107 31620769

[B45] Zhao S. P. Xu Z. S. Zheng W. J. Zhao W. Wang Y. X. Yu T. F.. (2017). Genome-wide analysis of the *RAV* family in soybean and functional identification of *GmRAV-03* involvement in salt and drought stresses and exogenous ABA treatment. Front. Plant Science. 8, 905. doi: 10.3389/fpls.2017.00905 PMC5459925 28634481

[B46] Zhao X. Y. Qi C. H. Jiang H. You C. X. Guan Q. M. Ma F. W.. (2019b). The MdWRKY31 transcription factor binds to the *MdRAV1* promoter to mediate ABA sensitivity. Horticulture Res. 6, 66. doi: 10.1038/s41438-019-0147-1 PMC6544635 31231524

[B47] Zhuang J. Sun C. C. Zhou X. R. Xiong A. S. Zhang J. (2011). Isolation and characterization of an AP2/ERF-RAV transcription factor BnaRAV-1-HY15 in *Brassica napus* l. HuYou15. Mol. Biol. Rep. 38, 3921–3928. doi: 10.1007/s11033-010-0508-1 21116861

