# Supplementary material for: Comprehensive analysis of cucumber RAV family genes and functional characterization of CsRAV1 in salt and ABA tolerance in cucumber
Source: Front Plant Sci. 2023 Feb 2;14:1115874. doi: 10.3389/fpls.2023.1115874 (PMC9933981; doi:10.3389/fpls.2023.1115874)
Supplement: Supplementary file 1 [file DataSheet_1.pdf]

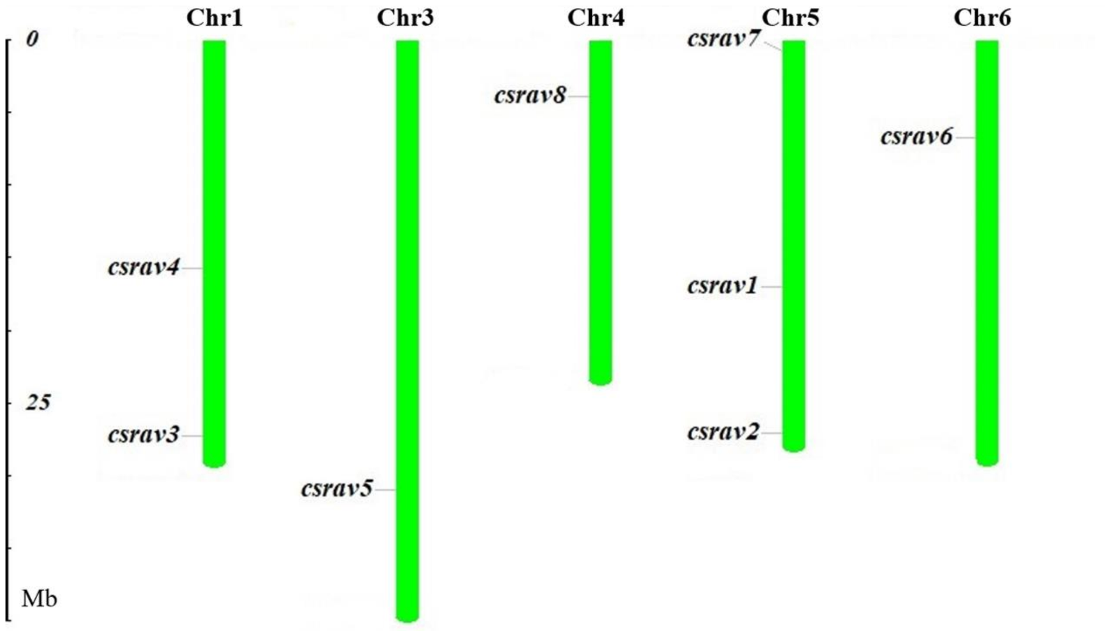

Supplementary Figure 1. The distribution of *CsRAV* family genes on chromosomes in cucumber.

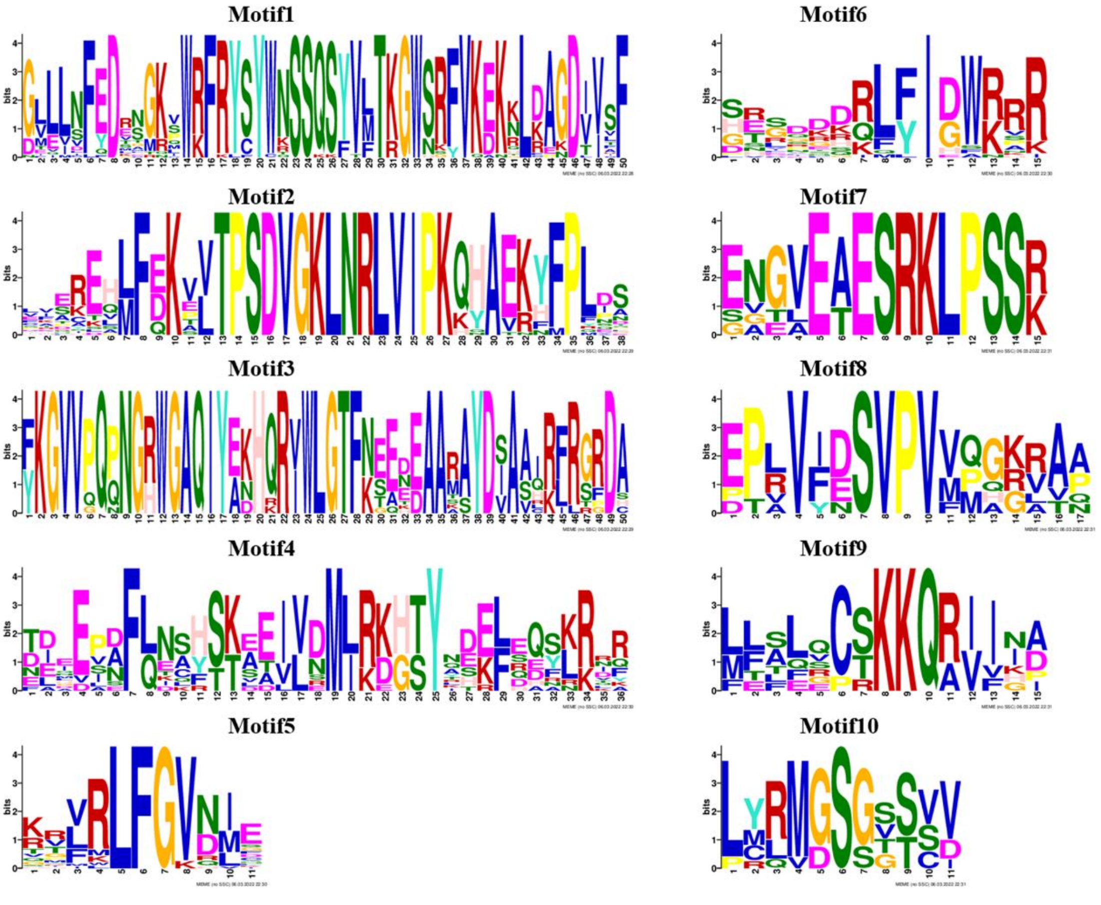

Supplementary Figure 2. The logos represented the ten conserved motifs of RAV

proteins.

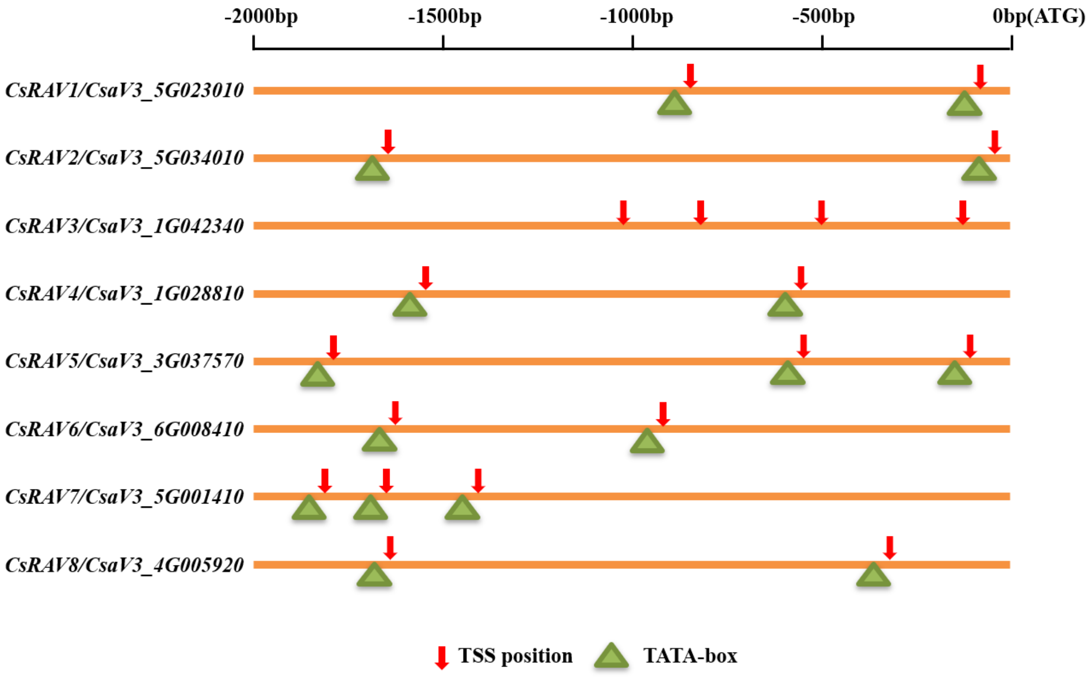

Supplementary Figure 3. The predictions of core elements including transcription start site (TSS) and TATA-box.

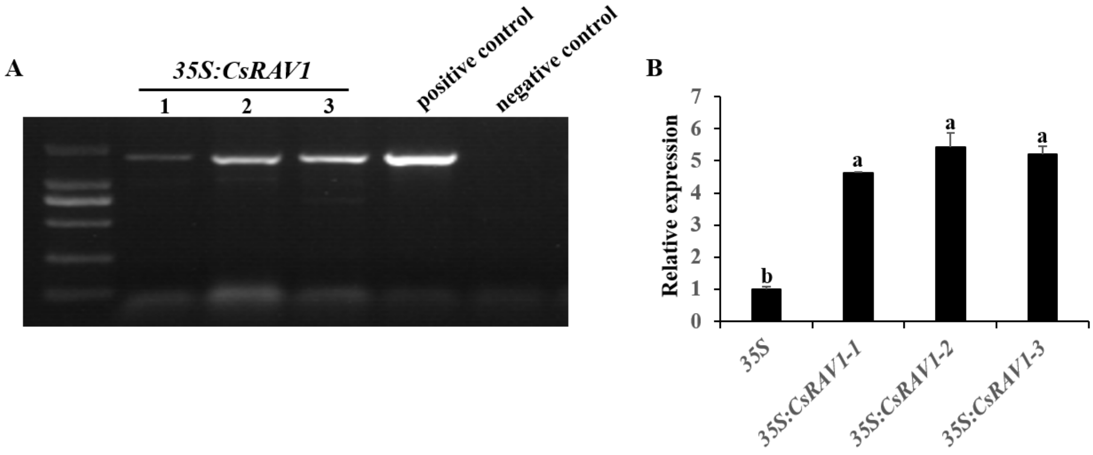

Supplementary Figure 4. Expression level of *CsRAV1* in its transgenic cucumber cotyledons. (A) The transgenic cucumber cotyledons were identified by PCR amplification. Positive control: *35S*:*CsRAV1* plasmid; Negative control: *35S* transgenic cucumber cotyledons. (B) The expression level of *CsRAV1* in *35S* and *35S*:*CsRAV1* (1, 2 and 3) transgenic cucumber cotyledons. The cucumber *β-actin* gene was used as internal control. Error bars were the standard errors (SE). Different letters indicated significant differences ($P < 0.05$).

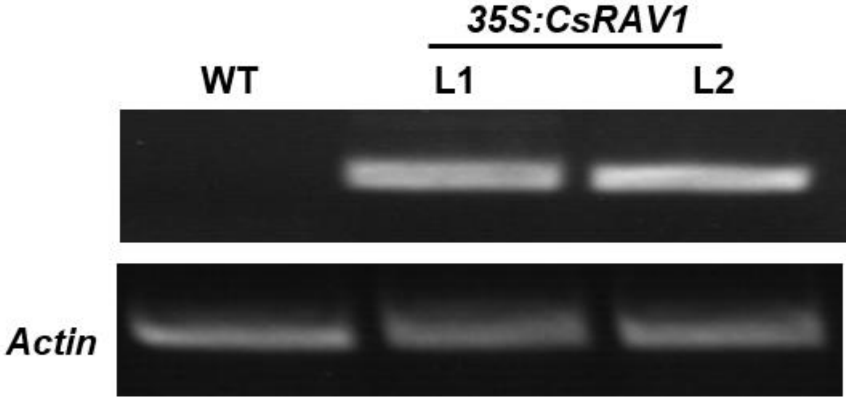

Supplementary Figure 5. *CsRAV1* transgenic plants were identified by semi-quantitative PCR.
